# Civility behaviour training programme and its influence on staff nurse organisational citizenship: a quasi-experimental study

**DOI:** 10.3389/fpubh.2025.1555198

**Published:** 2025-09-11

**Authors:** Eman Mohamed El-Shahat, Hemat Abdelazem Mostafa, Mona Mostafa Shazly

**Affiliations:** ^1^General Authority for Healthcare Accreditation and Regulation, Cairo, Egypt; ^2^Department of Nursing Administration, Faculty of Nursing, Ain Shams University, Cairo, Egypt

**Keywords:** nurse knowledge, civility behaviour, organisational citizenship behaviour, workplace, Egypt

## Abstract

**Background:**

This study aimed to assess the influence of a civility behaviour training programme on organisational citizenship among nurses in the workplace and to explore various predictors of nurses’ knowledge, workplace civility behaviour, and organisational citizenship behaviour.

**Methods:**

In 2023, a quasi-experimental study (one-group pre-test/post-test) was conducted. A convenience sample of 115 nurses was selected using the intervention method. The study was carried out at El-Sheikh Zayed Specialised Hospital, which is affiliated with the Ministry of Health and Population in Egypt. The self-administered questionnaires included (1) socio-demographic characteristics, (2) a questionnaire assessing nurses’ knowledge about civility behaviour, (3) a questionnaire assessing nurses’ levels of civility perception, and (4) a questionnaire assessing nurses’ levels of organisational citizenship behaviour (OCB) prior to the implementation of the training programme. Statistical analysis was performed using version 25 of SPSS.

**Results:**

A total of 115 nurses were included, with a mean age of 36.24 ± 5.9 years; of them, 59.5% were men, and 55% had 5–10 years of experience. The chi-square test showed highly significant relationships between the citizenship behaviour of nurses and their educational level and attendance at training courses, with *p*-values <0.01**. Additionally, there were significant correlations between total knowledge, perception related to workplace civility behaviour, and citizenship behaviour at pre-intervention, post-intervention, and at 3 months follow-up. The correlation coefficients indicate strong positive relationships (*r* = 0.569, *r* = 0.573, and *r* = 0.641) between perception and citizenship behaviour pre-intervention, post-intervention, and at the 3-month follow-up intervention, respectively.

**Conclusion:**

The implementation of the civility behaviour training programme had a significant positive impact on the organisational citizenship behaviours of the staff nurses. In addition, the majority of staff nurses had a high perception of workplace civil behaviour and citizenship behaviour after the training programme.

## Introduction

Civility behaviour (CB) is a polite act or expression toward others, and is a form of gracious goodness that contributes to mutual respect, effective communication, and collaboration (1). On the contrary, incivility behaviour is a set of discourteous behaviours that are intended to harm and are considered subversively deviant, undermining, or violating norms that lead to many undesirable consequences. These behaviours may occur in ignorance or oversight, and the intention of the perpetrator may be unclear. The behaviours are characterised as ambiguous, low in intensity, rude, discourteous, and displaying a lack of respect for others (1).

Incivility by nurses has increased over the last few years, with uncivil acts by nurses ranging from aggressive verbal confrontations to threats against physical safety, affecting both nurses and patients (2). Incivility in nursing is defined as rude or disruptive actions that can lead to many adverse effects, such as disengagement from various work activities, psychological or physiological distress for the individuals involved, and, if left unaddressed, can develop into threatening situations (2).

Civility should be prioritised and implemented as a foundational approach in the workplace. New nurses can be a factor in changing culture in practical settings. Managers must provide professional role modelling because it is critical to staff development and socialisation into the nursing profession. The professional role model includes attire, language, and communication. Effective communication can be modelled by using respectful language, maintaining inclusive attitudes, teaching and demonstrating civil discourse, and listening to staff. To be effective, role models must be aware of their own behaviour (3). The most essential strategies to promote CB within an organisation are showing respect for one another, taking civility reminders into the clinical setting, co-creating norms for clinical settings, discouraging gossip, speaking openly about the need for change, holding one another accountable, and dealing with issues before they become insurmountable (4, 5).

Organisational citizenship behaviour (OCB) is a term used to describe positive and constructive employee behaviours that are not part of their formal job description. OCB is a significant and critical part of any successful organisation that improves productivity with optimal use of available resources, promotes morale, fosters effective communication and cooperation between employees, and creates a positive working environment (6).

CB and OCB are closely related to each other. It has been demonstrated that employees who experience respect and civility at the workplace also own the duty of showing positive behaviour toward the organisation and coworkers. Such relations are developed on the basis of norms of reciprocity (1). Thus, both CB and OCB are fundamental in the nursing profession, given the frequent and wide-spectrum interactions nurses need to perform while communicating with peers and colleagues, physicians, managers, and patients and their families. They are essential for effective communication (7).

Nurses are on the front line of healthcare providers. Their attitudes and behaviours are fundamental to judging the quality of care provided (8). They need both civility and OCB in their practice to help create a comfortable work environment and improve work, as well as patient satisfaction. Such an environment promotes collaboration among healthcare providers, particularly between nurses and doctors, although there is a paucity of research addressing this issue (3).

As a result of CB within the organisation, the quality of social interactions at work improved and plays an important role in employee well-being at the workplace. However, positive social interactions raise job satisfaction and commitment, and the quality of work life. In addition, the member will be able to contribute to the well-being of another, helping others with appropriate and valuable information, optimal use of time, strong self-control and self-discipline, and encouraging a positive work environment and performance; all these previous issues fall under the concept of OCB (9). Furthermore, the relationship between the OCB of nurses and the CB in the workplace is vital to the success of healthcare organisations. Promoting a civility climate enhances collaboration, job performance, and overall organisational effectiveness by encouraging nurses to engage in beneficial extra-role activities. Studies consistently support the notion that a respectful and supportive work environment is fundamental to fostering OCB among nurses, ultimately leading to better patient care and a more cohesive healthcare team (10).

Workplace CB and OCB constitute an important part of the organisational climate perceived by nurses due to their positive impacts on work environments, with consequent positive outcomes on performance and care quality. Additionally, they are essential to attract new nurses to the profession and help them stay in the profession. Furthermore, if each nurse develops awareness of respectful behaviour and the necessary skills, it is anticipated that nurses will serve as role models and that these behaviours will spread throughout organisational healthcare and beyond. Therefore, the primary objective of this study was to assess the influence of a civility behaviour training programme on organisational citizenship behaviour among staff nurses. Additionally, the study explores various predictors of nurses’ knowledge, workplace CB, and OCB.

## Materials and methods

### Study design and period

A quasi-experimental study design (one-group pre-test/post-test) was conducted from April 2023 to September 2023 to assess the perception of the staff nurses about CB and its relationship to OCB and to explore various predictors of nurses’ knowledge, workplace CB, and OCB.

### Study setting

The study was carried out at El-Sheikh Zayed Specialised Hospital, which is affiliated with the Ministry of Health and Population in Egypt. The hospital has a single building with a capacity of 165 beds and consists of 8 floors. The study was carried out in the 21 units of the hospital.

### Study participants and sampling technique

A convenience sample of 115 nurses was recruited for this quasi-experimental study using the intervention method (the training programme in this study).

#### Training Programme planning

The CB programme was developed over six weeks (15 May to 30 June 2023) based on a comprehensive review of related literature using textbooks, scientific articles, and Internet searches. The researchers designed the CB programme for the staff nurses. The general objective of the programme was to improve the knowledge, attitudes, and practices of nurses about CB. Specific sessions included communication skills, conflict resolution, teamwork, and ethical decision making. The programme content was divided into theoretical and practical components, with each session incorporating case studies, role-playing activities, and group discussions to reinforce learning. In addition, teaching methods were identified, and the location to conduct the programme sessions was booked and prepared in collaboration with the nursing director and the director of training and development at El-Sheikh Zayed Specialised Hospital.

#### Implementation of the training programme

The programme was implemented over two months (1 July to 30 August 2023) and consisted of eight 2-h sessions per group, totalling 16 h of training. The participants were divided into four groups, two groups trained in parallel on Saturdays and Thursdays. Each group completed the programme in 1 month. The sessions were held in the hospital conference room and used a variety of teaching methods, including lectures, brainstorming, role playing, and group activities. The teaching medium included PowerPoint presentations, videos, posters, and flip charts. Participants received handouts summarising the content of the programme.

#### Post-programme evaluation

The researchers concluded the training programme by summarising its content and inviting nurses to share any questions or feedback in an open discussion forum. Subsequently, the staff nurses were asked to complete an immediate post-test questionnaire, using the same study tools used before the intervention. The post-test question was collected at the last session of the programme, so it was done at the end of August 2023.

#### Follow-up

A follow-up test was repeated 3 months after the implementation of the training programme at the end of August 2023, so it was done at the end of November 2023, using the same data collection tools.

### Eligibility criteria

All available nurses, both genders, aged 20 to 60 years, and who worked for at least two years in the settings mentioned above were included in the current study. On the contrary, volunteers and other healthcare providers were excluded.

### Data collection

In the current study, researchers distributed data collection tools to all staff nurses using self-administered questionnaires, including four tools: the first is an interview-based questionnaire to assess the sociodemographic characteristics of the nurses studied and the job characteristics of study subjects. The second is to assess the nurses’ knowledge regarding CB. The third is to assess the level of civility perception of nurses. The fourth is the level of OCB for nurses before implementing the training programme. These questionnaires were distributed three times throughout the study: before the programme, immediately after the programme, and three months post-implementation. The peer test was conducted from May 2023 until the end of June 2023. The nursing training programme was developed by the researchers after reviewing the literature to improve nurses’ perceptions of CB and OCB in the workplace (3, 11, 12). The implementation of the programme took 2 months, from the beginning of July 2023 to the end of August 2023. At the end of the last session, a post-test was conducted to assess the effect of the training programme, which took place at the end of August 2023. The follow-up phase was carried out three months after the implementation of the programme, at the end of November 2023, using the same evaluation tools.

### Part I: An interview-based questionnaire

This questionnaire was designed by the investigator based on a review of the related literature and was written in simple English to gather data on the sociodemographic characteristics of the nurses studied and the job characteristics of the study subjects, such as age, gender, marital status, nursing qualifications, years of experience, and previous attendance of related training courses.

### Part II: Nurses’ knowledge about the civility behaviour tool

In the current study, the nurse’s knowledge about the short form of the CB survey was used. This tool was designed by the researchers after reviewing related literature (13–15) to assess nurses’ knowledge about CB. It was written in English to collect data.

### Scoring system

The nurses’ knowledge about the CB questionnaire consists of 32 items: multiple-choice questions. These items cover topics such as the definition of civility, its benefits to nurses, other care providers, and patients; its relation to workplace safety; and the detrimental effects of incivility. A key response to the model was categorised on the basis of the correctness of the responses. Each correct answer received “two points” and incorrect answers received “one point.” These points were then summed and converted into percentage scores. This total score was 32, and the knowledge levels were classified into three categories: poor knowledge score < 60%, moderate percentage score of 60% to < 75%, and good knowledge score ≥ 75%.

### Part III: Nurse perception of workplace civility behaviour tool

In the current study, the short form of the CB survey was used to assess nurses’ perceptions of the workplace. This tool was developed by Clark (16) and adapted from previous studies (17–19) to assess the CB of staff nurses across various study phases.

### Scoring system

The nurse’s perception of the CB questionnaire in the workplace consists of 20 items. Participants rated the items using a five-point Likert scale from 1 = Never to 5 = Always. The options “Always” are considered positive responses, while “Never” is considered a negative response. The total scores of positive responses related to all domains are 100. This total score is classified into three categories: low percent score < 60%, moderate percent score from 60% to < 75%, and high percent score ≥ 75%.

### Part IV: Organisational citizenship behaviours tool

This self-report questionnaire was originally developed by Podsakoff et al. (20) and Podsakoff and Mackenzie (21) and modified by Eyupoglu (22). The researchers will adopt it to measure the level of OCB and evaluate the OCB of the staff nurses in the various phases of the study.

### Scoring system

In the current study, the OCB questionnaire consists of 24 items divided into five dimensions [Altruism (6 items), Conscientiousness (5 items), Sportsmanship (4 items), Courtesy (4 items), and Civic Virtue (4 items)]. Participants rated items using a five-point Likert scale from 1 = strongly agree to 5 = strongly disagree. The option “strongly agree” is considered a positive response, while “strongly disagree” is considered a negative response. The total scores of positive responses related to all domains are 120. This total score is classified into three categories: low percent score < 60%, moderate percent score from 60% to < 75%, and high percent score ≥ 75%.

### Content validity and reliability

The validity of the face and content of the study instruments, the knowledge of the nurse about the civility questionnaire at work, the perception of the CB questionnaire by the nurse at work, and the OCB questionnaire were determined by a panel of five experts in nursing administration, two professors from the department of nursing administration, Ain Shams University and three professors from the department of nursing administration at Cairo University. The experts revised the tools for clarity, relevance, completeness, easy language, and usability. Some changes were made while the final forms were developed. Since the original instruments were developed in English, cultural and linguistic adaptation was performed to ensure appropriateness for the Egyptian healthcare context. In addition, the reliability of the tools developed was tested using Cronbach’s alpha coefficients, demonstrating high internal consistency for the nurse’s knowledge about the workplace civility questionnaire 32 items, which was 0.906; the nurse perception of the workplace CB questionnaire 20 items, which was 0.874; and the OCB questionnaire 24 items, which was 0.913.

### Pilot study

A pilot study was carried out in April 2023, and was carried out in 10% of the sample size (12 nurses) who participated in a pilot study to evaluate the applicability, clarity, and effectiveness of the tools and to estimate the time to fill it, which ranged between about 20–30 min. No adjustments or changes were made, and nurses were added to the findings of the pilot study.

### Ethics statement

A formal approval was obtained from the Research Ethics Committee of the Faculty of Nursing, Ain Shams University, Registered number 24.12.421, to carry out the study. Additionally, written informed consent was obtained from the hospital nursing director and all participating nurses. Participants were informed of their right to withdraw from the study at any time without providing a reason.

### Data analysis

Statistical analysis was performed using SPSS version 25. Quantitative data are presented as mean ± standard deviation, whereas categorical variables are presented as percentages. An independent sample t-test was used to determine whether there was a significant difference between the means of the variables. Pearson’s correlation coefficient (r) was used to evaluate the relationships between variables. In this study, the chi-square test was used to analyse the significance of the difference between different categorical variables. ANOVA is a type of inferential statistic used to determine if there is a significant difference between the means of three groups. Where significant main effects were detected (*p* < 0.05), post hoc Tukey’s HSD tests were conducted to compare the three phases (pre, post, and follow-up phases). Cochran’s Q test was applied to determine if there are differences in a dichotomous dependent variable across three or more related groups. Additionally, multivariable linear regression models were used to model the relationship between a scalar response and one or more explanatory variables. The cutoff point used to determine statistical significance was set at a *p*-value less than 0.05, with *p* < 0.01 indicating a highly significant result.

## Results

### Socio-demographic characteristics of staff nurses

Table 1 reveals the socio-demographic characteristics of the studied staff nurses. A large percentage 43.4% of the nurses studied were in the age group 41–60 years, with a mean age of 36.24 ± 5.9 years; 59.5 and 67.8% of them were men, 82.7% of them were married, 41% of them had technical health institute education, 54.9% of them had 5–10 years of experience, with a mean age of 36.24 ± 5.9 years, 11.35 ± 7.8, and only a small percentage (11.6%) had attended training about workplace civility.

**Table 1:** Socio-demographic characteristics of staff nurses in the study.

Variables	*n* (%)
Age	
20–30	21 (17.9)
31–40	45 (38.7)
41–60	49 (43.4)
Mean ± SD	36.24 ± 5.9
Gender	
Men	69 (59.5)
Women	46 (40.5)
Marital status	
Single	20 (17.3)
Married	95 (82.7)
Nursing qualifications	
Nursing diploma	21 (18.5)
Technical health institute education	47 (41.0)
Bachelor’s degree	47 (40.5)
Work of experience (years)	
2–5 years	15 (13.3)
5–10 years	63 (54.9)
More than 10 years	37 (31.8)
Mean ± SD	11.35 ± 7.8
Attendance training about workplace civility	
Yes	13 (11.6)
No	102 (88.4)

Data are expressed as percentages for categorical variables. The chi-square test was used to examine differences in the prevalence of different categorical variables. A *P*-value less than 0.05 was considered statistically significant. SD, standard deviation; *n*, number; (%), percentage.

**Figure 1:**
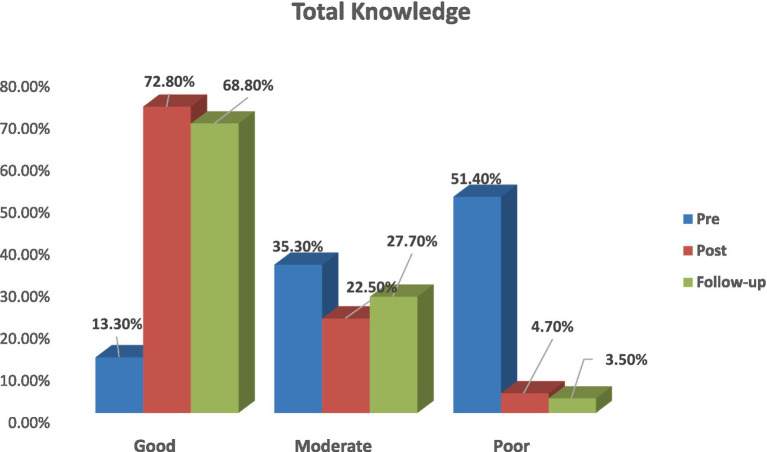
Total nurses’ knowledge about workplace civility behaviour throughout the intervention phases in the study setting.

Figure 1 shows that 51.4% of the staff nurses had poor knowledge pre-intervention; 72.8% of them had good knowledge post; and 68.8% of them had moderate knowledge at follow-up, with a highly significant difference at *p* < 0.01**.

### Nurses’ perception of workplace civility behaviour (CB)

Table 2 displays the distribution of the nurses’ perception scores in the context of workplace CB. The table indicates notable improvements post the intervention programme. A one-way repeated measures ANOVA demonstrated statistically significant differences across the three assessment phases. There was a significant rise in high perception scores from pre-intervention (10.4%, *n* = 12) to post-intervention (79.8%, *n* = 92; *p* < 0.001), with this gain being maintained at the 3-month follow-up (77.5%, *n* = 89; *p* < 0.001 compared to pre-intervention). There were no significant differences between post-intervention and follow-up scores (*p* = 0.600), demonstrating intervention effect sustainability. Consequently, low perception scores decreased significantly from pre-intervention (65.3%, *n* = 75) to post-intervention (4.0%, *n* = 4; *p* < 0.001) and remained low at follow-up (5.2%, *n* = 6). The mean perception scores showed a parallel improvement, increasing from 44.17 ± 12.7 pre-intervention to 88.42 ± 16.43 post-intervention, with sustained enhancement at follow-up (86.37 ± 19.3).

**Table 2:** Mean score of staff nurses’ perception related to workplace CB throughout intervention phases in the study.

Level of perception	Pre-programme phase	Post-programme phase immediately	Follow-up phase (after 3 months)	Statistical test *Post hoc* Tukey’s HSD
	*n* (%)	*n* (%)	*n* (%)	
High	12 (10.4)	92 (79.8)	89 (77.5)	P overall < 0.001 Pre vs. Post < 0.001 Pre vs. Follow-up < 0.001 Post vs. Follow-u*p* = 0.600
Moderate	28 (24.3)	19 (16.2)	20 (17.3)	
Low	75 (65.3)	4 (4.0)	6 (5.2)	
Mean ± SD	**44.17 ± (12.7)**	**88.42 ± (16.43)**	**86.37 ± (19.3)**	

Data are expressed as means ± SD for continuous variables. ANOVA with Tukey’s *post-hoc* test: all phase comparisons *p* < 0.001 except post-intervention vs. follow-up (*p* = 0.600). CB, civility behaviour. A *P*-value less than 0.05 was considered statistically significant. Highly statistically significant *p* < 0.001**. CB, civility behaviour; SD, standard deviation; *n*, number; (%), percentage. Low (<60% of total score), Moderate (60 to <75%), and High (≥75%). Bold values indicate the primary results of interest for each phase, representing the highest frequency (mode) category or the key mean value with its accompanying SD as highlighted by the authors. If multiple statistics are bolded in a row, they denote the focal point for that phase (e.g., the most prevalent perception level or the main summary statistic for that line).

### Overall impact of intervention on OCB dimensions

Table 3 shows statistically significant enhancement across all five OCB dimensions. Repeated one-way measures ANOVA combined with Tukey’s *post-hoc* tests revealed significant time effects for each OCB dimension (all *p* < 0.001), with large effect sizes (*η*^2^ > 0.14). In terms of altruism, it increased from 31.2 to 80.9% (*p* < 0.001). Moreover, courtesy improved from 27.2 to 75.1% (*p* < 0.001). Sportsmanship increased from 17.9 to 80.9% (*p* < 0.001). Similarly, civic virtue showed growth from 28.9 to 76.3% (*p* < 0.001). In the last dimension, conscientiousness showed a marked improvement (22.5 to 76.9%; *p* < 0.001), with mean scores increasing from 10.4 ± 1.9 to 23.4 ± 4.7. Maintenance of these improvements at the 3-month follow-up (all dimensions *p* < 0.001 vs. baseline) indicates sustainable intervention effects on OCB of nurses. These findings support existing evidence that targeted civility interventions can effectively enhance OCB among nursing professionals.

**Table 3:** Distribution of staff nurses related to OCB throughout the intervention phases (pre, post, and follow-up) in the study.

Dimensions of OCB	Level of perception	Pre-programme phase	Post-programme phase immediately	Follow-up phase (after 3 months)	Statistical test *Post-Hoc* Tukey’s HSD
		*n* (%)	*n* (%)	*n* (%)	
Altruism	High	36 (31.2)	93 (80.9)	85 (73.9)	*P* overall < 0.000 Pre vs. Post < 0.001 Pre vs. Follow-up < 0.001 Post vs. Follow-up < 0.001
	Moderate	19 (16.8)	15 (12.7)	20 (17.4)	
	Low	60 (52)	7 (6.4)	10 (8.7)	
	Mean ± SD	12.3 **±** (5.9)	29.35 **±** (5.9)	26.21 **±** (6.7)	
Courtesy	High	31 (27.2)	86 (75.1)	76 (66.5)	*P* overall < 0.000 Pre vs. Post < 0.001 Pre vs. Follow-up < 0.001 Post vs. Follow-up < 0.001
	Moderate	20 (17.3)	9 (7.6)	14 (11.5)	
	Low	64 (55.5)	20 (17.3)	25 (22.0)	
	Mean ± SD	9.81 **±** (2.7)	24.0 **±** (4.8)	21.4 **±** (5.8)	
Sportsmanship	High	21 (17.9)	92 (80.9)	80 (69.4)	*P* overall < 0.000 Pre vs. Post < 0.001 Pre vs. Follow-up < 0.001 Post vs. Follow-up < 0.001
	Moderate	26 (23.1)	12 (9.8)	17 (15.0)	
	Low	68 (59)	11 (9.3)	18 (15.6)	
	Mean ± SD	8.3 **±** (1.88)	21 **±** (4.3)	18.9 **±** (3.9)	
Civic virtue	High	33 (28.9)	88 (76.3)	77 (67.1)	*P* overall < 0.000 Pre vs. Post < 0.001 Pre vs. Follow-up < 0.001 Post vs. Follow-up = 0.665
	Moderate	15 (13.3)	9 (8.1)	12 (9.8)	
	Low	67 (57.8)	18 (15.6)	26 (23.1)	
	Mean ± SD	9.70 ± (3.4)	19.6 ± (3.6)	18.4 **±** (4.5)	
Conscientiousness	High	26 (22.5)	88 (76.9)	81 (70.5)	*P* overall < 0.000 Pre vs. Post < 0.001 Pre vs. Follow-up < 0.001 Post vs. Follow-up < 0.001
	Moderate	16 (13.9)	11 (9.2)	13 (11.0)	
	Low	73 (63.6)	16 (13.9)	21 (18.5)	
	Mean ± SD	10.4 **±** (1.9)	23.4 **±** (4.7)	21.3 **±** (4.2)	

Data are expressed as means ± SD for continuous variables. ANOVA with Tukey’s *post-hoc* test: all phase comparisons *p* < 0.001 except post-intervention vs. follow-up (*p* = 0.600). CB, civility behaviour. A *P*-value less than 0.05 was considered statistically significant. Highly statistically significant *p* < 0.001**. CB, civility behaviour; SD, standard deviation; *n*, number; (%), percentage. Low (<60% of total score), Moderate (60 to <75%), and High (≥75%).

### Correlations between nurses’ knowledge, perception, and OCB throughout the intervention phases

Table 4 demonstrates that there were strong and highly significant correlations between total knowledge, perception related to workplace CB, and OCB throughout the intervention phases. In the pre-intervention phase, the correlation coefficients indicate strong positive relationships between knowledge and OCB (*r* = 0.682, *p* < 0.001) and between CB perception and OCB (*r* = 0.569, *p* < 0001). Moreover, it shows that post-intervention, these correlations slightly increased, reflecting strengthened relationships between these variables. In the follow-up phase, it was demonstrated that the strong correlations persisted, with a slightly different magnitude. These findings demonstrate that an increase in nurses’ level of knowledge and perceptions of workplace civility improved and was associated with increased levels of OCB, underscoring the importance of educational and training interventions in enhancing organisational behaviour.

**Table 4:** Spearman correlation coefficient (r) between dimensions of perception related to workplace CB, and OCB of nurses pre-intervention, post-intervention, and follow-up intervention.

Phases (Dimensions)		Spearman’s rank correlation coefficient	
		Total knowledge	Perception-related workplace CB
Pre- intervention			
Perception-related workplace civility behaviour	*R*	0.503**	
	*P*-value	<0.001**	
Citizenship behaviour	*R*	0.682**	0.569**
	*P*-value	<0.001**	<0.001**
Post- intervention			
Perception-related workplace civility behaviour	*R*	0.586**	
	*P*-value	<0.001**	
Citizenship behaviour	*R*	0.690**	0.573**
	*P*-value	<0.001**	<0.001**
Follow-up intervention			
Perception-related workplace civility behaviour	*R*	0.490**	
	*P*-value	<0.001**	
Citizenship behaviour	*R*	0.586**	0.641**
	*P*-value	<0.001**	<0.001**

*Correlation is statistically significant at *p* < 0.05*.

**Correlation is highly statistically significant at *p* < 0.001**.

CB, civility behaviour; OCB, organisational citizenship behaviour.

### Predictors of nurses’ knowledge scores

Table 5 reveals that nursing experience and education level were significant predictors of knowledge scores. The model indicates that both factors had high positive significant coefficients (*p* < 0.01**) for education level and attended training courses, and also high negative significant coefficients (*p* < 0.05*) for experience. While there is no effect for age, gender, and marital status, *p* > 0.05. The studied factors contribute to a substantial portion of the variance in knowledge scores (R-square = 0.598, ANOVA *F* = 12.350, *p*-value 0.000**).

**Table 5:** Multiple linear regression model for the studied nurses’ knowledge score.

Items	Unstandardized coefficients		Standardized coefficients	*T*-test	
	*B*	Std. error		*t*	*P*-value
(Constant)	0.872	0.390		6.029	<0.01**
Age	−0.298	0.024	−0.189	1.293	>0.05
Gender	0.019	0.009	0.012	0.098	>0.05
Marital status	0.076	0.031	−0.054	1.005	>0.05
Nursing experience	−0.379	0.190	−0.309	3.906	<0.05*
Nursing qualifications	0.560	0.319	0.281	5.783	<0.001**
Attending training courses	0.493	0.286	0.316	6.702	<0.001**

The differences between means were tested by using an independent sample *t*-test. A *P*-value less than 0.05 was considered statistically significant. Highly statistically significant *p* < 0.001**. Unstandardised beta *B*. This value represents the slope of the line between the predictor variable and the dependent variable. R-squared = 0.598, ANOVA: *F* = 12.350, *p*-value < 0.001**.

### Predictors of workplace civility behaviour

To enhance the clarity and precision of the regression analyses presented in the manuscript, it is essential to provide a more detailed justification for testing each model independently. Testing each predictor separately, such as knowledge and perception, allows for a clearer understanding of their individual effects before assessing their combined impact. This approach helps to evaluate the strength of each variable as a factor that influences the outcome independently. Furthermore, the manuscript should address multicollinearity and the assumptions underlying the regression models. Specifically, the variance inflation factor (VIF) can be used to assess correlations between predictors, and other assumptions, such as linearity, normality, and homoscedasticity, should be tested using methods such as the Breusch-Pagan test. It is also crucial to explicitly clarify that knowledge and perception are hypothesised to be independent predictors of (OCB), thus establishing the analysis in a clear theoretical framework. After testing the predictors individually, it would be beneficial to run a combined model to assess the impact of these variables together, providing a more comprehensive understanding of their joint influence on OCB. Employing multivariate analysis or multiple regression models will enable a thorough investigation of this relationship. Finally, aligning these analyses with the research objectives will offer a more robust and cohesive explanation of how knowledge and perception contribute to OCB, thus strengthening the overall contribution of the manuscript to the field.

Table 6 reveals that nursing experience, education level, and age were significant predictors of civility behaviour. The model indicates that the level of education and the training courses attended had highly positive significant coefficients (*p* < 0.01**), and the nursing experience had negative significant coefficients (*p* < 0.05*). Age also showed a significant negative effect (*p* < 0.05*). Gender and marital status showed no significant effect (*p* > 0.05). The factors studied contributed to a substantial portion of the variance in the workplace CB scores (R-square = 0.603, ANOVA *F* = 16.809, *p*-value 0.000**).

**Table 6:** Multiple linear regression model for civility behaviour.

Items	Unstandardised coefficients		Standardised coefficients	*T*-test	
	*B*	Std. error		*t*	*P*-value
(Constant)	0.580	0.398		3.856	0.014*
Age	−0.386	0.059	−0.319	4.601	<0.05*
Gender	0.019	0.010	0.012	0.509	>0.05
Marital status	0.117	0.043	0.093	1.098	>0.05
Nursing experience	−0.391	0.068	−0.383	3.786	<0.05*
Nursing qualifications	0.467	0.145	0.509	6.102	<0.001**
Attending training courses	0.471	0.109	0.452	5.996	<0.001**

Differences between means were tested using an independent sample *t*-test. A *P*-value less than 0.05 was considered statistically significant. Highly statistically significant *p* < 0.001**. Unstandardised beta B; this value represents the slope of the line between the predictor variable and the dependent variable. R-squared = 0.598, ANOVA: *F* = 12.350, *p*-value < 0.001**.

### Predictors of Organisational citizenship behaviour

Table 7 shows the factors affecting that affect OCB using a multiple linear regression model. The results indicate that the level of education and the training courses attended were significant predictors of OCB, with high positive significant coefficients (p 0.01**). Gender showed slightly significant negative coefficients (*p* < 0.05*). Age, experience, and marital status had no significant effects (*p* > 0.05). The model explains a substantial portion of the variance in citizenship behaviour scores (R-square = 0.603, ANOVA *F* = 28.463, *p*-value 0.000**).

**Table 7:** Multiple linear regression model for organisational citizenship behaviour.

Items	Unstandardised coefficients		Standardised coefficients	*T*-test	
	*B*	Std. error		*t*	*P*-value
(Continuous)	0.599	0.238		4.675	0.019*
Age	0.145	0.023	0.198	2.106	>0.05
Gender	−0.348	0.025	0.318	4.098	0.021*
Marital status	0.109	0.024	0.059	0.794	>0.05
Nursing experience	0.167	0.018	0.102	1.026	>0.05
Nursing qualifications	0.498	0.380	0.301	6.098	<0.001**
Attending training courses	0.501	0.406	0.346	7.908	<0.001**

Differences between means were tested using an independent sample *t*-test. A *P*-value less than 0.05 was considered statistically significant. Highly statistically significant *p* < 0.001**. Unstandardised beta *B*; this value represents the slope of the line between the predictor variable and the dependent variable. R-squared = 0.603, ANOVA F = 28.463, *p*-value = 0.000**.

## Discussion

In our study, the researchers discussed important points related to assessing the nursing knowledge regarding civility behaviour (CB) pre-programme and post-programme, assessing nursing perception regarding CB pre-programme and post-programme, assessing organisational citizenship behaviour (OCB) among staff nurses pre- and post-programme, and evaluating the effectiveness of implementing CB training programmes on staff nurses’ OCB. Moreover, the researchers hypothesised that the implementation of the CB training programme would affect staff nurses’ OCB, representing the aim of this study. Regarding the socio-demographic characteristics of the studied nurses, the current study revealed that less than half of them were in the age group of 41–60 years, with a mean age of 36.24 ± 5.9 years. This finding is consistent with Hossny et al. (23), who reported that the mean age of the studied nurses was 33.61 ± 8.69 years old. The majority of the participants were married. Moreover, half of the studied group had 5–10 years of experience, while less than one-third of them had attended training programmes about workplace civility. This study was in agreement with Hossny and Sabra (3) and with Abd-Elrhaman and Ghoneimy (24), who noted that most of the nurses studied were married. The predominance of male nurses, coupled with the fact that most of them were married, may reflect societal or cultural dynamics that affect workplace interactions and civility. Almost half of the studied group were males, with more than 5 years of experience, and about one-third of them had attended training programmes on workplace civility. In contrast, Gazica and Spector (25) found that most of their studied group had not attended training programmes and that many had around 5 years of experience. On the other hand, the current study result was in disagreement with Phan and Hampton (26), who conducted the study and reported that the majority of study subjects were women.

According to the total knowledge of the nursing staff about CB in the workplace throughout the intervention phases, the current study results displayed that more than half of staff nurses had poor knowledge pre-intervention, while less than three-quarters of them had good knowledge post-test and more than two-thirds at follow-up, with a highly significant difference.

This could be related to only a small percentage having attended training about workplace civility pre-intervention. Moreover, the majority of them had more than 5 years of work experience, which reflects positively on their change level in the workplace CB. The current study results were in agreement with Elsayed et al. (27), who mentioned that more than two-thirds of nurses had a low level of knowledge in the pre-test. While at the immediate post-test, the majority of them had a high level of knowledge. After three months of programme implementation, there were statistically significant differences in knowledge scores compared with prior assessments (*p* < 0.05).

Regarding the mean score of staff nurses’ perception related to workplace CB throughout intervention phases, the current study results confirmed the changes in perceptions related to workplace civility behaviours. Mean scores improved significantly from the pre-intervention to post-intervention and follow-up phases, reflecting enhanced perceptions of CBs such as assuming goodwill, respectful communication, avoiding gossip, and mentoring others. These changes are statistically significant.

The findings suggest that workplace environments where nurses feel encouraged to practice and reinforce CB may contribute to improved perceptions. The data indicate that effective leadership promotes a positive civil environment by implementing policies, communication strategies, and team-building initiatives that foster respect and collaboration. This result was in agreement with Alam et al. (28), who reported that there was significant improvement in the studied group scores regarding their perception of workplace CB post-programme implementation, with a statistically significant difference (*p*-value <0.001). Moreover, the present study findings were in agreement with previous studies by Abd-Elrhaman and Ghoneimy (24) and with Biomy et al. (29), who demonstrated that there was a highly statistically significant improvement in staff nurses’ level of workplace civility after implementation of the programme. Additionally, the present study findings were similar to Hossny and Sabra (3), who showed that the majority of the studied nurses had a high perception level of workplace civility climate post the intervention, with a highly positive statistically significant correlation. On the other hand, the current study results were contrasted with Clark (30), who stated that the majority of the participants had a high perception level of workplace civility without any education sessions about workplace civility.

Regarding the distribution of the nurses studied in relation to total OCB throughout the intervention phases, the present study indicated that approximately three-quarters of nurses had a high level of OCB post-intervention and follow-up phases, while one-quarter had a low level of organisational citizenship pre-intervention. This finding is consistent with Alamelu et al. (31) and with Peng (32), who clarified that there was a notable improvement in the percentage of the nurses studied who showed high OCB post-intervention, with a statistically significant difference pre-programme and post-programme implementation. The present results were similar to the study by Abo Baraka (33), who found that a positive workplace civility climate was significantly correlated with higher levels of innovative work behaviour. This is consistent with the findings of our initial study, which indicated a dramatic increase in nurses’ perception of civility post-intervention. The study concluded that fostering a civil workplace environment could improve nurses’ innovative capabilities, supporting the idea that interventions to improve civility can produce positive outcomes in nurse behaviour and performance.

Concerning the relationships between total knowledge, perception related to workplace CB, and OCB, the current study found moderately stronger post-intervention correlations between knowledge, workplace CB perceptions, and OCB. This study suggests a synergistic relationship. The findings suggest that improved understanding of workplace civility appears to foster more positive perceptions, which, in turn, may motivate nurses to demonstrate increased organisational citizenship behaviours. These results align with theoretical framework models that improved knowledge and positive perceptions create environments conducive to voluntary workplace contributions.

The results of the present study were in agreement with Elsayed et al. (27), who reported that there is a statistically significant (positive) correlation between leadership competencies and both the civility climate of work and mental well-being for the data, and also between the civility climate of work and the mental well-being scales. Furthermore, the present study results are in harmony with the study in Indiana by Howard and Embree (34) and another study in Malaysia by Liu (35), who showed that there was an improvement in perception scores level with significance for the experimental group postintervention. In contrast, WCI scores decreased significantly for the control group. Within the experimental group, all participants noted the successful use of a positive conflict management strategy after the educational intervention. On the other hand, the results of the current study were in the opposite line to the study in the Netherlands by Der-Kinderen et al. (36), who found that there was no statistically significant relationship between the civility climate in the workplace and the participation in work among the subjects studied.

Depending on the factors influencing knowledge scores, using a multiple linear regression model, the results of the current study revealed that nursing experience and level of education were significant predictors of knowledge scores. The model indicates that the education level and attendance at training courses had highly positive significant coefficients, while nursing experience had a significantly negative coefficient. There is no effect for age, gender, and marital status. The factors studied contributed to a substantial portion of the variance in knowledge scores.

The findings suggest that these components may be particularly important for improving knowledge outcomes. The data indicate that without continuous education or recent training, experienced nurses may have less exposure to newer developments in CB and workplace practices, which could explain the negative association between experience and knowledge scores. Furthermore, the results imply that demographic factors appear to play a limited role in influencing nurses’ knowledge levels. Rather, knowledge enhancement appears to be more strongly associated with educational background and ongoing training opportunities than with personal demographic characteristics. This study aligns with Armstrong (37), who showed that education is imperative to fight and eliminate incivility in nursing by educating about incivility in a meaningful way. Furthermore, this finding was consistent with Oppel and Mohr (38), who revealed that there is a positive association between civility climate and civility toward patients with respect to patient experience outcomes, while there is no effect for age, gender, and marital status.

Regarding the factors affecting OCB through a multiple linear regression model, the results of the present study found that the level of education and the attendance at training courses were significant predictors of OCB, with highly positive significant coefficients. Gender showed slightly negative significant coefficients. Age, experience, and marital status had no significant effects. The model explains a substantial portion of the variance in citizenship behaviour scores. This emphasised that nurses with higher education and more training are likely better equipped to exhibit behaviours that align with citizenship principles, such as cooperation, helping others, and upholding professional standards. In addition, the slightly negative significant coefficient for gender may reflect differences in how OCB is perceived or implemented among male and female nurses. It is possible that gender norms or expectations in the workplace influence the way these behaviours are expressed, although the exact reasons would require further investigation.

The present results were similar to the study in Pakistan by Jamal and Siddiqui (39), who revealed that age, experience, and marital status had no significant effects. Moreover, gender showed slightly negative significant coefficients in the OCB scores. On the other hand, the results of the current study were in contrast to the study in India by Gupta and Singh (40), who revealed that gender showed slightly positive significant coefficients; also, age and years of experience had direct significant effects on OCB scores.

## Strengths and limitations

The main strength of our study was that it is one of the first studies to examine staff nurses’ knowledge and perceptions about CB and its relation to OCB in Giza, specifically in El-Sheikh Zayed City. However, the study has several limitations. First, the use of a convenience sampling approach may limit the representativeness of the sample, which should be acknowledged as a constraint. Second, this study was conducted in a single hospital under the Ministry of Health and Population, which may affect the generalisability of the findings. Third, the absence of a control or comparison group restricts our ability to determine the effects of the intervention. Future studies should involve multiple hospitals in the Cairo governorate to improve the applicability of the findings.

## Conclusion

The current study concluded that implementing a civility behaviour training programme can positively impact organisational citizenship among nursing staff. By promoting respectful and professional interactions, such programmes can foster a more positive work environment and encourage nurses to engage in extra-role behaviours that benefit the organisation. However, the effectiveness of these programmes may depend on factors such as the specific content and delivery of the training, as well as the organisational culture and support for civility.

## Data Availability

The original contributions presented in the study are included in the article/supplementary material, further inquiries can be directed to the corresponding author.
